# Birth of the Society of Divorees – Changing Patterns of Civil Status in Later Life

**DOI:** 10.1093/geroni/igab046.3322

**Published:** 2021-12-17

**Authors:** Torbjorn Bildtgard, Peter Öberg

**Affiliations:** 1 Stockholms University, Stockholm, Stockholms Lan, Sweden; 2 University of Gävle, Gävle, Gavleborgs Lan, Sweden

## Abstract

Half a century ago Lopata used the concept “society of widows” to describe the gendered reality of late life singlehood, where widowed women were excluded from coupled social life, depended on a community of other widows for social integration, and refrained from initiating new relationships due to “sanctification” of their former husbands. We use Swedish, American and EU census data and a national survey to Swedes 60-90 years old (n=1225; response rate 42%) to illustrate a substantial change in the demographic landscape of late life singlehood. More people enter later life as divorcees or become divorced at a high age. Among Swedes 60+ divorcees outnumber widowed people, and the incidence of late life divorce has more than doubled since the millennium in what has been called the “grey divorce revolution”. Many other Western countries follow the same demographical trend, posing important questions about the transformation of late life singlehood. Based on two Swedish studies we will show that the structure of the late life single community is becoming less gender skewed as a consequence of the emerging society of divorcees, and that in this society relationship careers are increasingly complex, attitudes to repartnering increasingly liberal and partner sanctification seldom an issue. We conclude by proposing the concept “society of divorcees” for this new demographic landscape of late life singlehood, argue that research is needed to capture this new reality, and discuss the implications of this change for access to social support later life.

